# Predictability of In-Office SureSmile^®^ Clear Aligners: A Retrospective Analysis of Anterior Tooth Movements

**DOI:** 10.3390/dj14060370

**Published:** 2026-06-15

**Authors:** Gina Marie Georgi, Jesper Delfs, Cita Nottmeier, Bärbel Kahl-Nieke, Maija Eltz, Matthias Sonnleitner, Till Koehne, Carmen U. Schmid-Herrmann

**Affiliations:** 1 Department of Orthodontics, University Medical Center Leipzig, Liebigstraße 12, 04103 Leipzig, Germany; 2 Private Orthodontic Practice Kiefer & Zahn, Kücknitzer Hauptstraße 2, 23569 Lubeck, Germany; 3 Department of Orthodontics, University Medical Center Hamburg-Eppendorf, Martinistraße 52, 20246 Hamburg, Germany; 4 Private Orthodontic Practice Eltz Institut für Kieferorthopädie, 1010 Vienna, Austria; 5 Private Orthodontic Practice Ruperti-Kieferorthopädie, Rottmayrstraße 3, 83410 Laufen, Germany

**Keywords:** aligner treatment accuracy, SureSmile, anterior tooth movement

## Abstract

**Background:** Aligner workflows range from outsourcing the design and manufacturing process to in-office scanning, planning, and fabrication. This exploratory retrospective study aimed to evaluate whether discrepancies between planned and achieved anterior tooth movements with SureSmile aligners remain within predefined clinical thresholds. **Methods:** A total of 21 dental arches from 14 patients who underwent SureSmile aligner treatment with in-office fabricated aligners were retrospectively analyzed. Digital models of the planned setup were superimposed on models of the clinically achieved outcomes to assess the accuracy of anterior tooth movements by comparing planned and clinically achieved outcomes. **Results:** Rotational movements showed larger discrepancies than translational movements. Mean discrepancies for angulation and axial rotation were 3.42 ± 1.50° and 4.50 ± 2.12°, respectively, both exceeding the predefined clinical threshold of 2°. In contrast, translational discrepancies remained below the clinical threshold of 0.5 mm, with mean values of 0.17 ± 0.11 mm for mesiodistal, 0.27 ± 0.16 mm for labiolingual, and 0.38 ± 0.17 mm for vertical movements. Translational discrepancies were significantly lower than the predefined clinical threshold in patient-level analyses, whereas rotational discrepancies were not. **Conclusions:** In-office SureSmile aligner workflows showed acceptable accuracy for translational anterior tooth movements, whereas angular movements frequently exceeded predefined clinical thresholds.

## 1. Introduction

In the era of digital orthodontics, the value of a treatment plan lies in its clinical deliverability. Understanding how predictably appliances realize planned movements is essential for indication, overcorrection strategies, and patient counseling.

Orthodontic tooth movement with custom-made clear aligners has become integral to routine clinical practice [1], not only among specialists but increasingly within general dentistry, driven by patient demand and the accessibility of digital workflows [1,2].

Among the available systems, SureSmile (Dentsply Sirona, Bensheim, Germany) offers a fully digital workflow that allows clinicians to independently define treatment objectives and staging parameters while maintaining full control over the aligner workflow. This in-office solution enhances flexibility and cost-efficiency, particularly in small-practice settings.

SureSmile was selected because it provides a standardized in-office aligner workflow with an integrated, software-generated attachment proposal. In the present study, the automatically generated attachment design was reviewed and approved by an experienced orthodontist without manual modification. This predominantly software-guided approach reduces operator-dependent variability and allows a focused evaluation of planned versus achieved tooth movements under clinically realistic conditions. Despite widespread adoption of aligner therapy, the predictability of specific tooth movements, particularly anterior movements such as tipping, rotation, and extrusion, remains subject to debate [3,4]. Although these movements have been investigated extensively, reported outcomes vary substantially across studies due to methodological and system-related differences. While systematic reviews report good outcomes in mild-to-moderate malocclusions, they also highlight considerable heterogeneity due to differences in software platforms, materials, and movement staging protocols [5,6,7]. In addition, differences in reported aligner effectiveness may be influenced by variations in staging strategies, which are often insufficiently described or standardized across studies and therefore represent an additional source of heterogeneity.

Sorour et al. [8] emphasized the need to interpret simulation discrepancies within the context of clinical relevance, defining deviations greater than 0.5 mm for translational movements and greater than 2° for rotational movements as clinically meaningful.

Against this background, and given the persistent limitations of clear aligner therapy in achieving bodily movement, rotational control of globular teeth, and predictable vertical movement [1,3,4,9,10], the aim of this study was to evaluate the accuracy of mild anterior tooth movements by comparing planned tooth movements with clinically achieved outcomes using in-house aligners designed with the SureSmile software (version 7.6).

## 2. Methods and Materials

### 2.1. Study Design

This was a retrospective, exploratory, single-center analysis of consecutive patients treated with in-office SureSmile clear aligners. The study was performed in accordance with the Declaration of Helsinki and reported in line with the STROBE guidelines. All analyses were performed on fully anonymized patient data. The Ethics Committee of the Hamburg Medical Association confirmed that the project did not constitute research involving human beings under Section 9 (2) of the Hamburg Chamber Act for the Medical Professions and did not require consultation (reference 2024-300432-WF). Informed consent for the use of clinical records for research purposes was obtained from all subjects.

### 2.2. Patient Selection

**Figure 1:**
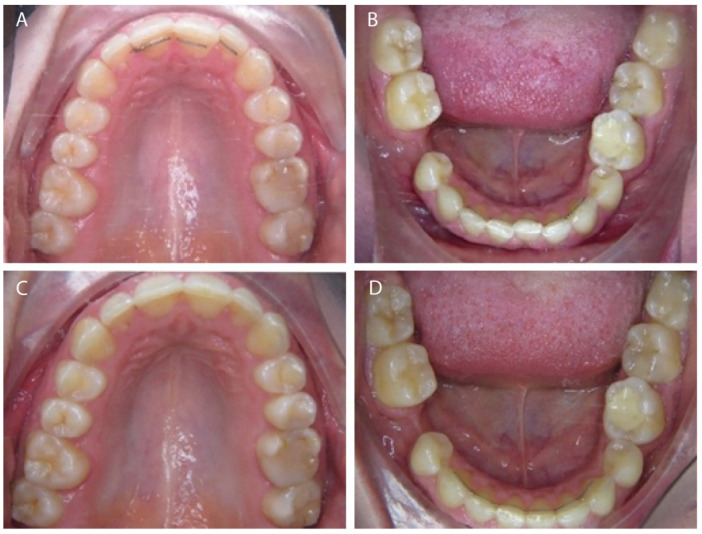
Exemplified pre- and posttherapeutic situations: pretherapeutic crowding in the anterior region of the maxilla (**A**) and mandible (**B**); posttherapeutic situation of the maxilla (**C**) and mandible after alignment (**D**).

**Figure 2:**
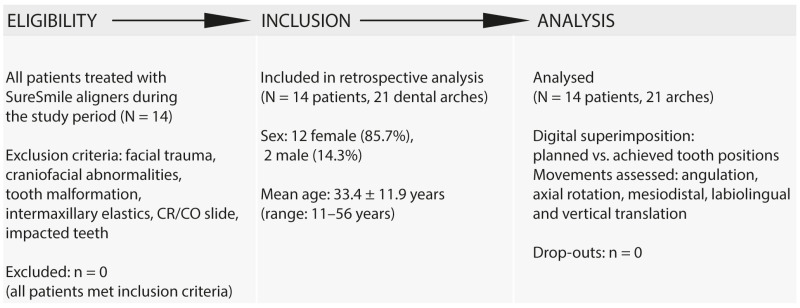
Study flow diagram in accordance with STROBE reporting guidelines.

Patients who had undergone SureSmile aligner treatment in an orthodontic office were retrospectively investigated (Figure 1). The cohort comprised both patients seeking primary orthodontic correction and patients presenting with post-retention relapse following previous orthodontic treatment. In the relapse subgroup, the reasons included fixed retainer fracture or partial debonding and reduced compliance with removable retainer wear. Inclusion required complete pre- and post-treatment intraoral scans for each patient. Patients were excluded if they had a history of facial trauma, craniofacial abnormalities, tooth malformation, or impacted teeth. Patients requiring intermaxillary elastics (Class II, Class III, settling, or criss-cross) or presenting with a centric relation/centric occlusion slide were also excluded in order to evaluate the aligner effect in isolation and avoid additional forces or occlusal repositioning unrelated to the planned aligner-driven movement (Figure 2).

### 2.3. Treatment Planning and Aligner Fabrication

All treatments were planned and clinically supervised by a single experienced orthodontist throughout the study. The digital setup was planned within the SureSmile software environment and subsequently reviewed and approved by an orthodontist with clinical experience in aligner therapy. SureSmile automatically determined the number of aligners as well as the attachment size and positioning based on the planned tooth movements. The software-generated attachment configuration was reviewed but not manually modified in the analyzed cases. After transmission of the STL staging model files, these were printed with a 3D printer (Form 2, Formlabs, Berlin, Germany) using a resin (Dental Model Resin, Formlabs, Berlin, Germany). The aligners were subsequently fabricated at the Eltz Institute in Vienna, Austria.

Transparent aligners (0.75 mm) (EssixAce, Dentsply Sirona, Bensheim, Germany) were thermoformed on the printed models using a pressure thermoforming unit (Biostar^®^, Scheu Dental, Iserlohn, Germany). After trimming of the aligner margins and quality control by an orthodontist, the aligners were delivered to the patient.

### 2.4. Aligner Protocol and Patient Compliance

The patients were asked to wear the aligners for at least 22 h a day and to remove them only for eating, tooth brushing, and cleaning the aligners. A wearing time of two weeks per aligner was recommended. All patients were strongly advised that a reduced wearing time could lead to quality losses. Follow-up visits at the orthodontic office took place every six weeks.

In all cases, approximal enamel reduction was necessary to create space. This was done prior to the insertion of the respective aligner as defined by the treatment planning. The mean number of aligners required was 8.8 aligners per jaw per patient.

### 2.5. Data Acquisition and Superimposition Protocol

**Figure 3:**
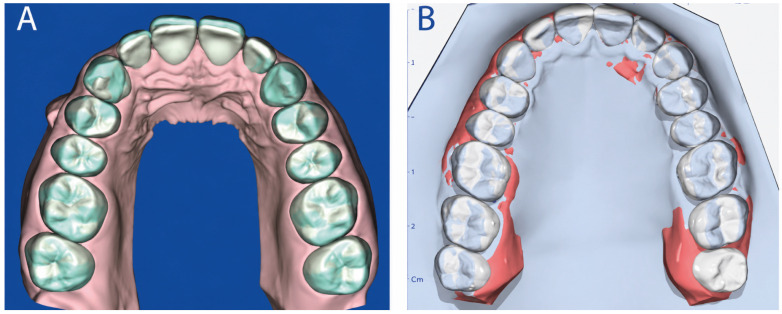
(**A**) User interface of SureSmile showing the upper arch of the initial situation (turquoise) and the setup (white); (**B**) dental superimposition in OnyxCeph of planned posttherapeutic outcome (segmented model; red/white) and situation at the end of treatment (grey/light blue).

Two superimpositions were performed for each case: (i) the planned setup was superimposed onto the initial (pre-therapeutic) scan to quantify the planned tooth movement, and (ii) the post-therapeutic scan was superimposed onto the planned setup to quantify the remaining discrepancy between planned and achieved tooth positions. The superimposition was performed using the OnyxCeph Inspect 3D module (version 3.2.174, Image Instruments, Chemnitz, Germany) (Figure 3). For this purpose, the crowns of the setup were segmented after the orientation of the jaws in the coordinate system. The OnyxCeph Inspect 3D module applies a deterministic iterative closest-point registration on the segmented stable posterior teeth; the operator-dependent component is therefore limited to the crown segmentation step, which was performed by a single trained operator following a standardized protocol.

The stationary posterior teeth (premolars and molars), which could be identified based on the setup, were used as superimposition structures. Reciprocal effects on the posterior reference teeth were considered negligible, as no posterior movements were planned, the anterior corrections were of small magnitude, and any reciprocal force component was distributed across at least four stable reference teeth. Subsequently, OnyxCeph was able to calculate values for both the planned movements and the movements actually performed.

### 2.6. Classification of Tooth Movements

The following movement types were considered: angulation (mesiodistal tipping), axial rotation, mesiodistal translation, labiolingual translation, and vertical translation (intrusion/extrusion). Due to the low number of teeth (≤3) with planned inclination (labiolingual tipping), this movement type was excluded from further analysis.

### 2.7. Measurement of Effectiveness

All distance and angular measurements were derived by the OnyxCeph Inspect 3D module after segmentation of the individual tooth crowns. Translational movements were referenced to the geometric centroid of the segmented crown, and angular movements were calculated relative to the long axis of the segmented crown.

The effectiveness of the respective tooth movement was examined separately for each anterior tooth type (maxillary central incisor, maxillary lateral incisor, mandibular central incisor, and mandibular lateral incisor). To determine a value for effectiveness, a ratio between performed and planned movement was created. For this purpose, the planned distance (pr = difference between set-up and initial model) and the remaining distance (rr = difference between set-up and final model) were recorded. The effective distance results from the difference between pr and rr (er = pr − rr).

$$effectivity=\frac{pr-rr}{pr}$$

The resulting value is a direct measure of implementation effectiveness. To quantify movement implementation, the ratio between achieved and planned movement was calculated. A value of 100% indicates full implementation of the planned movement. Values exceeding 100% represent an overshoot of the planned movement, whereas values below 0% indicate movement occurring in the opposite direction relative to the planned movement vector. Distances were interpreted relative to the direction of the planned movement, not as signed Euclidean distances. Mean values and standard deviation were calculated using GraphPad Prism (version 10.5.0, GraphPad Software, San Diego, CA, USA). The terms ‘discrepancy’ (absolute difference between planned and achieved movement, in mm or °) and ‘effectiveness’ (ratio between achieved and planned movement, in %, also reported as ‘accuracy’ in the tables) are used consistently throughout the manuscript. Negative effectiveness values were retained in the analysis to preserve directional information and avoid overestimating mean effectiveness.

### 2.8. Clinical Relevance Thresholds

According to the American Board of Orthodontics (ABO) guidelines [11], a marginal ridge discrepancy of 0.5 mm corresponds to a crown-tip deviation of approximately 2 degrees in an average-sized molar. Based on this, Sorour et al. [8] defined deviations >0.5 mm (translational) and >2° (rotational) as clinically relevant in their assessment of simulation efficacy. The ABO criteria were originally developed for the occlusal assessment of posterior teeth, not specifically for anterior aligner biomechanics, and are used here as the best available clinical reference values in the absence of established anterior-specific thresholds.

Following this rationale, the present study adopts the same thresholds to differentiate clinically meaningful discrepancies from minor deviations that are unlikely to influence treatment outcomes.

### 2.9. Statistical Analysis

To account for the clustered data structure, in which multiple teeth are measured within the same patient, the patient was defined as the statistical unit of analysis for all inferential testing. For each movement type, tooth-level absolute discrepancies between planned and achieved movements were calculated and averaged per patient, yielding one composite value per patient. Tooth-level results are additionally reported in descriptive tables to characterize movement behavior across individual tooth types. One-sample *t*-tests were performed to assess whether mean patient-level discrepancies were significantly lower than the predefined clinical thresholds. The tests were conducted as one-sided comparisons because the clinical hypothesis was directional, namely, whether discrepancies remained below the predefined thresholds. Normality of the patient-level mean discrepancies was inspected visually, and the *t*-test was retained as the primary analysis given its robustness with small samples and consistency with comparable studies in the field. A formal equivalence or non-inferiority framework was not applied because no clinically established equivalence margin exists for aligner-related tooth movement discrepancies; the 0.5 mm and 2° values were therefore used as descriptive clinical reference thresholds rather than as formal equivalence margins. Statistical significance was set at *p* < 0.05. All analyses were performed using R (version 4.5.2, R Foundation for Statistical Computing, Vienna, Austria).

## 3. Results

### 3.1. Discrepancies in Angulation and Axial Rotation

Angulation discrepancies were greater in the lower arch, with mean discrepancies of 6.20° for the lower central incisors and 6.60° for the lower lateral incisors (Table 1). The upper central and lateral incisors showed lower discrepancies of 3.40° and 3.03°.

The largest rotational discrepancies were found in the upper arch, with 11.10° for the upper central incisors and 6.04° for the upper lateral incisors. Lower incisors showed smaller discrepancies of 5.53° (central) and 1.98° (lateral). Only the lower lateral incisor remained within the clinical threshold of less than 2°, although this finding was accompanied by a large standard deviation (9.23°) (Table 1).

### 3.2. Discrepancies in Linear Translation

Regarding linear translation, nearly all mean values remained within the clinically acceptable threshold of <0.5 mm (Table 1). Exceptions were observed in the mesiodistal translation discrepancies of the upper central incisors (0.51 mm) and in the vertical translation discrepancies of the lower lateral incisors (0.51 mm). All other mesiodistal translation discrepancies ranged from 0.07 mm to 0.11 mm, labiolingual translation discrepancies from 0.02 mm to 0.27 mm, and vertical translation discrepancies between 0.09 mm and 0.38 mm (Table 1).

### 3.3. Accuracy of Individual Tooth Movements

In terms of translational movements, labiolingual translation demonstrated the highest overall accuracy at 100.93%, followed by mesiodistal translation at 68.16%. Vertical translation showed the lowest translational accuracy at 51.45% (Table 2).

Across individual teeth, the highest rotational accuracy was observed in the lower lateral incisor for rotation (83.59%), while the lowest was found in the upper central incisor for rotation (40.85%). Labiolingual translation in the upper lateral incisor reached the highest accuracy overall (114.66%). The lowest vertical accuracy was recorded in the lower central incisor (24.45%). Among the rotational movements, angulation showed the lowest overall accuracy with a mean of 43.81%, while axial rotation reached 52.67% (Table 2). Individual teeth with negative effectiveness values were observed, particularly for rotational and vertical movements, indicating movement opposite to the planned vector. Values exceeding 100% represent slight overcorrection in the intended direction and remained within tenths of a millimeter, well below the predefined clinical threshold of 0.5 mm.

### 3.4. Comparison to Clinical Thresholds

Patient-level mean absolute discrepancies for each movement type are summarized in Table 3.

For rotational movements, mean discrepancies exceeded the predefined clinical threshold of 2°. Angulation showed a mean discrepancy of 3.42 ± 1.50° (95% CI: 2.27–4.58°), while axial rotation reached 4.50 ± 2.12° (95% CI: 3.28–5.72°). One-sample *t*-tests indicated that these values were not significantly lower than the predefined threshold (*p* = 0.9893 and *p* = 0.9997, respectively).

In contrast, translational movements remained below the clinical threshold of 0.5 mm. Mesiodistal discrepancies averaged 0.17 ± 0.11 mm (95% CI: 0.09–0.25 mm), and labiolingual discrepancies averaged 0.27 ± 0.16 mm (95% CI: 0.18–0.35 mm). Vertical discrepancies averaged 0.38 ± 0.17 mm (95% CI: 0.28–0.48 mm). For all translational movements, the mean discrepancy was significantly lower than the predefined clinical threshold (*p* < 0.001 for mesiodistal and labiolingual movements; *p* = 0.014 for vertical movements). It should be noted that the low absolute discrepancies for vertical translation occurred against a background of small planned vertical movements; this finding reflects the limited magnitude of the planned correction rather than high vertical predictability per se, as also indicated by the corresponding effectiveness value of 51.45% (Table 2).

## 4. Discussion

This study highlights the fundamental limitations of aligner-based tooth movement, demonstrating that while certain translational movements can be achieved predictably, rotational corrections remain inherently less reliable.

Axial rotation and angulation frequently exceeded the predefined thresholds, which may indicate that planned overcorrection could be considered to achieve clinically acceptable outcomes.

The limited accuracy of angular movements observed in this study, particularly the large rotational discrepancies in the upper incisors (11.10° and 6.04°) and the pronounced angulation errors in the lower incisors (6.20° and 6.60°), is consistent with established biomechanical limitations of clear aligner therapy related to force transmission and moment control. Prior studies have shown that rotations and torque are inherently difficult to express because thermoformed plastics provide insufficient moment-to-force ratios and lose force rapidly during wear [4,9,12]. Taken together, these mechanisms help explain why nearly all angular discrepancies in our study exceeded the 2° clinical threshold, whereas translational movements remained within 0.5 mm. In contrast, the high accuracy of labiolingual translation (100.93%) and mesiodistal translation (68.16%) aligns with prior findings that linear translations are more predictably transmitted through aligner materials due to simpler force systems [13]. Some of the smallest translational discrepancies reported here are in the range of tenths of a millimeter and may approach the order of magnitude of the expected method error in crown-based superimposition; these values should therefore be interpreted with corresponding caution.

Although the deviations in vertical translation remained within the clinical threshold of <0.5 mm (*p* = 0.014), vertical translation showed the lowest effectiveness among all translational movements. This was particularly evident in the central incisors, which demonstrated low effectiveness values (30.71% and 24.45%), reflecting the well-documented difficulty of achieving predictable vertical translation with aligners [14,15,16]. Possible explanations proposed in the literature include unintended intrusive forces acting on the molars [17,18] as well as the so-called “watermelon seed effect,” in which deformation of the aligner generates forces that lead to unintentional intrusion of the displaced tooth [19]. The fact that vertical discrepancies remained within the clinical threshold despite an overall effectiveness of only 51.45% should not be interpreted as good vertical predictability: it reflects the small magnitude of the planned vertical movements in this cohort rather than a reliable implementation of vertical corrections. Larger planned vertical movements would be expected to produce proportionally larger absolute discrepancies. Furthermore, differences in the magnitude of planned vertical movements between upper and lower incisors could have influenced the absolute discrepancies, as larger planned movements tend to accumulate greater deviations [14,20]. Clinically, the limited predictability of vertical control in the upper arch has been linked to factors such as the bite-plane effect of the aligner [21] and the biomechanical difficulty of applying sustained intrusive forces in the upper arch without auxiliary anchorage [17].

For cases requiring large rotational corrections or substantial vertical movements, the use of SureSmile aligners may need to be considered with caution, as reduced predictability of these movements has been reported, and excessive deviations from the planned movement magnitude may compromise aligner seating and treatment outcome [22,23].

Mesiodistal movements were more predictably achieved in this cohort, although some deviations from the planned movement magnitude were observed (mean effectiveness 68.16%). Labiolingual translation showed the highest effectiveness in our dataset, with a mean value of 100.93%.

In line with these findings, Sorour et al. demonstrated that the clinical threshold of 2° for angular movements could not be maintained with either the Invisalign or Flash system, reinforcing the difficulty of achieving precise angular corrections reported for different aligner systems. When patients choose aligner therapy for reasons of comfort and aesthetics [24,25], the SureSmile aligner system represents a suitable option for managing mild to moderate anterior crowding [26]. In the context of in-house aligner production, SureSmile offers a highly standardized and guided planning workflow with fixed and case-related costs but comparatively lower technical demands on the clinician, whereas software solutions such as OnyxCeph (Modul Aligner 3D) enable more flexible and cost-efficient in-house aligner planning but require greater user input. Given the reduced predictability observed for most movement types, deviations from the planned movement magnitude should be anticipated during treatment planning. Overall, these findings suggest that SureSmile can provide clinically acceptable outcomes for selected indications, while highlighting the importance of recognizing the inherent mechanical limitations of aligner systems and incorporating them into individualized treatment planning. These findings should be interpreted as preliminary and specific to the in-office SureSmile workflow evaluated here. They may not generalize to outsourced SureSmile aligners, other thermoforming materials, different staging protocols, or workflows in which the software-generated attachment design is manually modified by the clinician [8].

## 5. Limitations

This study has several limitations. Posterior teeth were used as superimposition reference structures; while no active posterior movements were planned, minor reciprocal effects within the closed aligner system cannot be excluded. No formal intra-examiner reliability analysis or repeated measurements were performed, and the method error of the crown segmentation step was not quantified. The smallest translational discrepancies should therefore be interpreted with caution.

The clinical thresholds applied are derived from the ABO grading system, originally developed for posterior teeth; anterior-specific thresholds have not yet been established in the literature. Wear compliance relied on patient self-reporting, as objective monitoring was not available in this retrospective design. The sample of 14 patients from a single center without a priori power calculation limits generalizability; findings should be interpreted as descriptive of a specific in-office workflow rather than broadly representative. Finally, attachment placement was software-generated without manual modification, which reflects a deliberate standardization of the workflow but limits conclusions about the influence of clinician-driven attachment design on treatment outcomes.

## 6. Conclusions

The SureSmile aligner system showed acceptable accuracy for mild-to-moderate translational anterior tooth movements, whereas angular corrections and vertical movements were less predictable. Modest overcorrections may be considered for these movement types, and careful case selection is recommended when larger rotational or vertical changes are planned. SureSmile can be a suitable option for mild-to-moderate anterior crowding when its biomechanical limitations are taken into account during treatment planning. These preliminary, workflow-specific findings should be confirmed in larger prospective studies.

## Figures and Tables

**Table 1:** Mean values and standard deviations of rotational and translational discrepancies for the central and lateral incisors in both the maxillary and mandibular arches.

Tooth	Rotational Movements		Translational Movements		
	Angulation (°)	Axial Rotation (°)	Mesiodistal (mm)	Labiolingual (mm)	Vertical (mm)
Central incisor upper jaw	3.40 ± 8.02	11.10 ± 6.56	0.51 ± 0.10	0.21 ± 0.79	0.09 ± 0.57
Lateral incisor upper jaw	3.03 ± 5.55	6.04 ± 8.75	0.11 ± 0.56	0.24 ± 0.43	0.38 ± 0.47
Central incisor lower jaw	6.20 ± 0.57	5.53 ± 9.05	0.08 ± 0.46	0.27 ± 0.85	0.22 ± 0.62
Lateral incisor lower jaw	6.60 ± 1.23	1.98 ± 9.23	0.07 ± 0.69	0.02 ± 0.72	0.51 ± 0.42

**Table 2:** Accuracy (ratio between the planned tooth movement and the clinically achieved movement) in percent for every rotation and translation.

Tooth	Rotational Movements		Translational Movements		
	Angulation (%)	Axial Rotation (%)	Mesiodistal (%)	Labiolingual (%)	Vertical (%)
Central incisor upper jaw	50.1	40.85	64.46	105.16	30.71
Lateral incisor upper jaw	35.72	51.80	67.84	114.66	63.70
Central incisor lower jaw	58.75	43.88	68.87	83.22	24.45
Lateral incisor lower jaw	42.84	83.59	78.72	101.04	88.86
**Total Accuracy**	**43.81**	**52.67**	**68.16**	**100.93**	**51.45**

**Table 3:** Patient-level mean absolute discrepancies for rotational and translational movements relative to predefined clinical thresholds. Values are presented as mean ± SD and 95% CI. Absolute discrepancies between planned and achieved movements were calculated per tooth and averaged per patient. One-sample *t*-tests evaluated whether discrepancies were lower than the clinical thresholds (2° for angular movements, 0.5 mm for translational movements).

	Rotational Movements		Translational Movements		
	Angulation (°)	Axial Rotation (°)	Mesiodistal (mm)	Labiolingual (mm)	Vertical (mm)
mean discrepancy	3.42 ± 1.50	4.50 ± 2.12	0.17 ± 0.11	0.27 ± 0.16	0.38 ± 0.17
95% CI	2.27 – 4.58	3.28 – 5.72	0.09 – 0.25	0.18 – 0.35	0.28 – 0.48
*p*-value	0.9893	0.9997	<0.001	<0.001	0.014

## Data Availability

The data presented in this study are available on request from the corresponding author. The data are not publicly available due to privacy and ethical restrictions.
